# Glutamate Efflux across the Blood–Brain Barrier: New Perspectives on the Relationship between Depression and the Glutamatergic System

**DOI:** 10.3390/metabo12050459

**Published:** 2022-05-20

**Authors:** Benjamin Fredrick Gruenbaum, Alexander Zlotnik, Amit Frenkel, Ilya Fleidervish, Matthew Boyko

**Affiliations:** 1 Department of Anesthesiology and Perioperative Medicine, Mayo Clinic, Jacksonville, FL 32224, USA; bengruenbaum@gmail.com; 2 Department of Anesthesiology and Critical Care, Soroka University Medical Center, Ben–Gurion University of the Negev, Beer-Sheva 8410501, Israel; alexander.zlotnik.71@gmail.com (A.Z.); frenkela@clalit.org.il (A.F.); 3 Department of Physiology and Cell Biology, Faculty of Health Sciences and Zlotowski Center for Neuroscience, Ben–Gurion University of the Negev, Beer-Sheva 8410501, Israel; ilya@bgu.ac.il

**Keywords:** blood–brain barrier, blood glutamate scavengers, depression, glutamate

## Abstract

Depression is a significant cause of disability and affects millions worldwide; however, antidepressant therapies often fail or are inadequate. Current medications for treating major depressive disorder can take weeks or months to reach efficacy, have troubling side effects, and are limited in their long-term capabilities. Recent studies have identified a new set of glutamate-based approaches, such as blood glutamate scavengers, which have the potential to provide alternatives to traditional antidepressants. In this review, we hypothesize as to the involvement of the glutamate system in the development of depression. We identify the mechanisms underlying glutamate dysregulation, offering new perspectives on the therapeutic modalities of depression with a focus on its relationship to blood–brain barrier (BBB) permeability. Ultimately, we conclude that in diseases with impaired BBB permeability, such as depression following stroke or traumatic brain injury, or in neurogenerative diseases, the glutamate system should be considered as a pathway to treatment. We propose that drugs such as blood glutamate scavengers should be further studied for treatment of these conditions.

## 1. Introduction

Major depressive disorder (MDD) is a significant contributor to the development of disability [1]. Projections by the World Health Organization predict that depression will be the highest ranked cause of disease burden in middle- and high-income countries by the year 2030 [1,2]. In the United States, the prevalence of depression has been estimated at 17% [3]. Nearly two dozen distinct antidepressant medications involving monoaminergic systems are in use today [4]. These medications, though, have limited effectiveness, with many failing to provide long-term remission for patients [5]. Often, treatments do not begin to work until weeks or months of administration [6]. Side effects of antidepressants are common and significantly reduce the quality of life of patients [4,7]. New antidepressants that work quickly and effectively are necessary, especially for patients whose depression is not fully mitigated by traditional monoaminergic antidepressants. Conditions that have depression as a co-morbidity include subarachnoid hemorrhage, acute ischemic stroke, intracerebral hemorrhage (ICH) or traumatic brain injury (TBI), as well as chronic neurological conditions, such as Parkinson’s disease, Alzheimer’s disease, epilepsy, multiple sclerosis and schizophrenia.

Recent studies have reported on a promising new group of drugs called glutamate-based antidepressants, which have the potential to act in ways that traditional antidepressants cannot. In this review, we provide evidence for the involvement of the glutamate system in the development of depression. We specifically examine the disruption of the glutamate efflux through the blood–brain barrier (BBB). The purpose of this manuscript is to evaluate the glutamate theory and offer new perspectives on the therapeutic modalities of depression with a focus on BBB permeability.

## 2. Glutamate

Glutamate, a non-essential amino acid, is responsible for the majority of fast excitatory transmission in the brain, and another amino acid neurotransmitter, γ-aminobutyric acid (GABA), enables the majority of fast inhibitory transmission [8]. Data indicate that glutamate neurons and synapses represent the largest neurotransmitter system in the brain, besides the GABAergic system [8]. Glutamate has numerous biological functions and plays a critical role in cellular metabolism and synaptic plasticity [9,10]. Therefore, the brain is heavily regulated by glutamatergic excitatory functions, including by the GABAergic inhibitory component and neurons that release other types of neurotransmitters such as monoamines [8]. Monoamines, which regulate chemical neurotransmission, have been shown to regulate certain brain functions including sleep/wakefulness, hypothalamic activities, emotional/motivational activities mediated by limbic circuitry and value-based behaviors in the neocortex [11]. Changes in excitatory transmission that are offset by the inhibitory transmissions are the primary factors in these functions, especially in cognition and emotion.

## 3. Glutamatergic Hypothesis of Depression

A relationship between depression and glutamate has been firmly established for several decades [8,12,13]. The hypothesis about this relationship first emerged in the early 1990s, when antagonists of the N-methyl-D-aspartate (NMDA) ionotropic glutamate receptor were associated with antidepressant-like behaviors in mice [12]. In addition, infusion of low-dose ketamine, an NMDA receptor antagonist, was associated with substantial decreases in depressive symptoms in a human study [13]. Other glutamate receptors, including α-amino-3-hydroxy-5-methyl-4-isoxazolepropionic acid (AMPA), have become known as well.

More recent data continues to advance the idea that glutamate dysregulation plays a role in the biological mechanisms underlying depression [14]. Studies in animals and humans clearly show the involvement of the glutamate system in the pathophysiology of depression [8,12,13]. Several studies observed changes in glutamate levels in the blood, cerebrospinal fluid (CSF) and brains of patients with MDD. Higher glutamate levels were observed in the serum [15] of patients with MDD, and antidepressant treatment reduces those levels [16,17]. Increased glutamate levels have also been seen in the plasma of patients with MDD [18]. Depressive patients have higher glutamate levels in CSF [19] and in the frontal cortex, according to postmortem brain samples. A study by Mitani et al. determined that the relationship between plasma levels of glutamate and severity of depression was predicated on plasma levels of glutamate, in addition to levels of alanine and L-serine, as an indication of the severity of the disease [18].

The results of a recent meta-analysis of magnetic resonance spectroscopy (MRS) also support the theory of the important role of glutamatergic neurotransmission in the pathophysiology of depression [20]. Many studies indicated disrupted glutamate receptors (NMDA receptors, AMPA receptors and metabotropic glutamate receptors, or mGluR) in postmortem brain samples from individuals with MDD. In addition, positron emission tomography studies identified a reduction in the density of mGluR subtype 5 in patients with depression, which was also corroborated by a post-mortem study [21].

A reduction in the relative density of one form of a NMDA subunit in bipolar disorder and depressed patients is consistent with significant decreases in NMDA receptor density in the same series of cases, and the report of a decrease in NMDA receptor density in the frontal cortex of suicide victims [22]. An in situ hybridization study of patients with MDD showed significantly lower mRNA levels of subunits of NMDA receptors and AMPA receptors in the perirhinal cortex [23], though mRNA was not reduced in the hippocampus or entorhinal cortex. Western blotting analyses showed decreased levels of these same subunits of NMDA receptors in the anterior prefrontal cortex of patients with MDD [24]. Contradictorily, however, receptor binding assays and autoradiography in patients with MDD showed more binding of [3H]CGP39653 to a glutamate site on NMDA receptors in the hippocampus, though not in the entorhinal or perirhinal cortex [23]. Similarly, two recent reports demonstrated elevated levels of mGluR2/3 protein [25] and reduced levels of mGluR5 protein [26], which are both subtypes of mGluRs, in the prefrontal cortex of patients with MDD. Data from the selective mGluR5 antagonist [^11^C]ABP-688 and positron emission tomography indicate reductions in mGluR5 binding in multiple areas of the frontal, temporal and parietal cortices of patients with MDD [21]. In another meta-analysis, Luykx et al. [27] also noted region- and state-specific alterations in concentrations of glutamate and glutamine, an essential amino acid that is a result of glutamate conversion, in depression. The importance of glutamatergic neurotransmission in depression is also based on studies that showed altered glutamine concentrations despite normal glutamate levels [28,29].

As a whole, these data suggest that glutamate receptor abnormalities and changes in blood, plasma and brain glutamate levels are involved in the pathophysiology of MDD. Studies of depressive-like behavior in genetic models of deficient glutamate function and advances in the treatment of MDD with glutamate-based antidepressants strongly support this theory [30,31,32].

## 4. Depression and the BBB

Based on this theory of the involvement of glutamate in the development of depression, an increase in the concentration of brain glutamate is a significant factor in the development of depression. Here, we identify some potential mechanisms that lead to elevated brain glutamate levels. Some evidence shows that depression is a consequence of a destruction of the integrity of the BBB through its disturbance of the glutamate efflux.

Normal equilibrium of BBB glutamate is required to maintain an appropriate concentration gradient [33]. Under normal conditions, glutamate cannot move between the intraparenchymal and blood compartments of the BBB without adverse effects. The BBB consists of a physical barrier that defends the central nervous system (CNS) against influx of toxic substances in the blood. It can present high electrical impedance (≈2000 Ω/cm^2^), making it impermeable to ions [34]. The BBB is formed by brain microvascular endothelial cells and junctional complexes, an endothelial basement membrane and astrocyte end feet around the endothelial cells.

The BBB has distinct layers, each of which assists in restricting the flow of solutes [35]. Under physiological conditions, a high concentration of blood glutamate needs to penetrate at least five of these layers to enter the brain. Glutamate is transported at a relatively low rate, lower than other amino acids, from the blood into the brain [36]. Therefore, in normal circumstances, only a very minute amount of blood glutamate crosses the BBB into the brain [37].

There is a high range of sensitivity of nervous tissue to high concentrations of glutamate. However, there is evidence that a prolonged increase of even 10% of extracellular brain glutamate, i.e., glutamate neurotoxicity, can initiate non-degenerative processes that eventually lead to the development of depression [38].

Depression is a known co-morbidity of many conditions [39,40]. Given the theory of glutamate involvement in the development of depression and the importance of BBB permeability in these disorders, it is reasonable to consider that there is a link between depression and BBB permeability in these neurological and neurogenerative diseases.

In this review, we hypothesize that a moderate but prolonged increase of glutamate in the extracellular brain due to BBB disruption is the underlying mechanism of the development of depression when it occurs after stroke, after traumatic brain injury and in conditions with compromised BBB integrity associated with age, environment, or chronic disease.

## 5. The Imbalance of Blood-Glutamate Concentration Gradient Following Brain Injury

Many studies have found that during acute brain injury, such as subarachnoid hemorrhage, acute ischemic stroke, intracerebral hemorrhage (ICH) or traumatic brain injury (TBI), glutamate levels in the brain and blood can rise to very high levels [33]. This elevation has also been observed in patients with chronic neurological conditions, such as Parkinson’s disease, Alzheimer’s disease, epilepsy, multiple sclerosis and schizophrenia. These patients have significantly elevated glutamate levels in the blood and CSF compared to non-affected individuals, and the intraparenchymal-blood glutamate concentration gradient also becomes extremely high. Similarly, a recent study has proposed a connection between the gut–brain axis and glutamate that can contribute to the development of depression during gut–brain dysfunction [28].

A decrease may occur in the transport of intraparenchymal glutamate by the glymphatic system after brain insult. Studies have examined the role of regulators, especially aquaporin-4 (AQP4), and glutamate transporter-1 (GLT-1), which are the two essential water and glutamate astrocyte buffering pathways to the brain. In several different animal models of brain insult, including models of AD [41], aging [42], epilepsy [43] and ICH [44], AQP4 expression is lessened and its polarity becomes disrupted.

**Figure 1:**
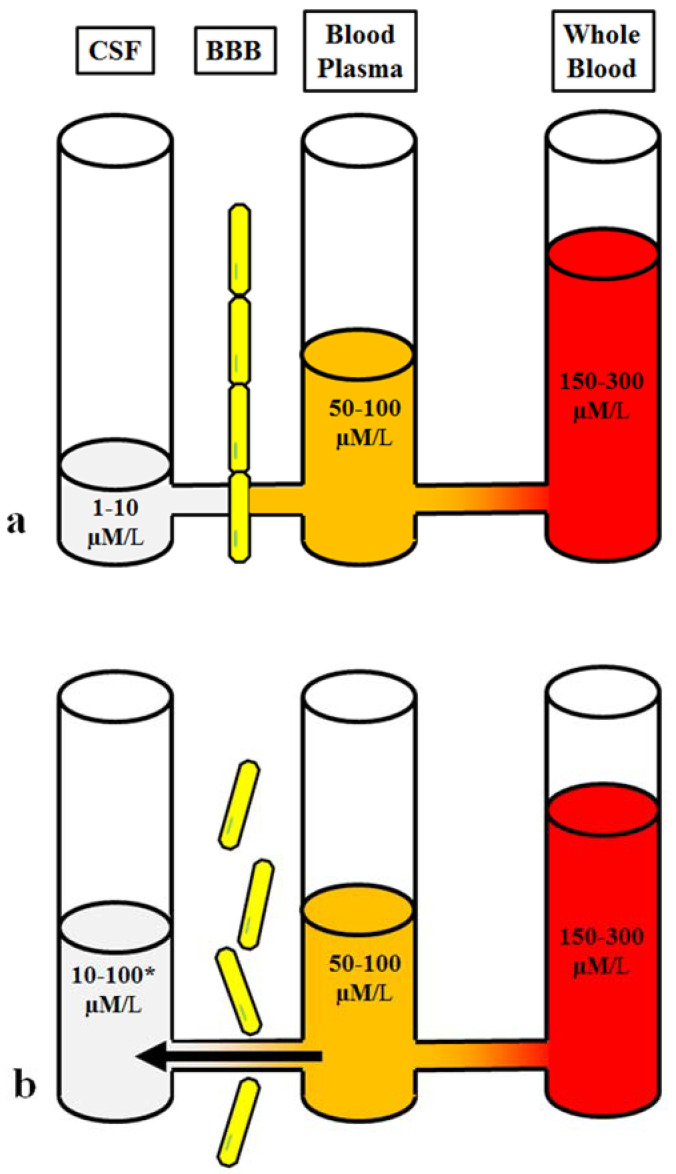
Brain-blood glutamate equilibrium: (**a**). Homeostasis of the normal physiological state. (**b**). Imbalanced glutamate under pathological conditions and disruption of BBB integrity. 10–100*µM/L The concentration of brain glutamate depends on the degree of disruption of BBB integrity.

Animal studies using dye injections [45] and magnetic resonance imaging (MRI) in humans [46] have also shown increased permeability of the BBB after a brain injury. These studies indicate that more blood glutamate can also cross into the brain through the functionally impaired BBB and have an effect on the increased intraparenchymal glutamate observed after brain injury (shown in Figure 1).

Until recently, doubts persisted about whether the BBB exhibits severe physical impairment following brain injury for a significant period of time. Studies have shown that recovery of BBB integrity can take 1–3 months [47] or even up to 10 months [48] in rat models, and in humans, recovery can take years [49]. Immediately after TBI, the concentration of cerebral glutamate increases, then decreases. However, the level does not decrease to normal levels, and the excess can last for many months and even years [50,51].

As noted above, damage to the BBB prevents effective clearance of cerebral glutamate from extracellular fluid into the bloodstream, in which excitatory amino acid transporters (EAAT) on endothelial cells propel the maintenance of the intraparenchymal-blood glutamate concentration gradient [33]. Thus, controlling excess glutamate is a primary goal in preventing the spread of brain damage. Extracellular glutamate is removed by several types of EAATs located on neurons and astrocytes. Loss of astrocytes and neurons as a result of the traumatic impact subsequently reduces the number of available sodium-dependent astroglial glutamate GLT-1 and EAATs, exacerbating the accumulation of extracellular glutamate [52].

## 6. Blood as the Source of Elevated Brain Glutamate

In healthy adults, the glutamate concentrations in the plasma and whole blood are 50–100 µM/L and 150–300 µM/L, respectively [53]. In several in vitro studies using acute brain slices, extracellular glutamate levels were recorded between 25 and 90 nM [54,55]; however, most in vivo studies using microdialysis found much greater increases in glutamate levels in the brain between 0.2 μM and approximately 20 μM [56,57]. Currently, researchers estimate that glutamate concentrations in the CSF or brain intercellular fluid range from 1 to 10 μM [58,59]. In the healthy brain, the glutamate concentration is much higher in the plasma, which in turn contains higher concentrations that the CSF, with a difference of 50 μM between these different approaches; this results in the intraparenchymal-blood glutamate concentration gradient [58]. Intraparenchymal glutamate homeostasis mainly depends on the integrity of the BBB, and its ability to control the influx of blood glutamate, and on the activity of endothelial glutamate transporters (EAATs), which constantly move intraparenchymal glutamate into the blood [60].

When restoring an imbalance in intraparenchymal-blood glutamate homeostasis, it is necessary to lower the elevated glutamate levels in both the blood and the brain. This process involves impeding the entry of blood glutamate into the brain and improving the transport efficiency of glial and endothelial EAATs and the glymphatic system under pathological conditions.

Glutamate does not solely exist in the brain or blood. One study showed that an increase in the glutamate concentration from 1 to 500 μM in the carotid artery in primary hypertension rats was associated with a higher rate of glutamate penetration into the brain [61]; additionally, systemic injection of glutamate has been indicated to aggravate brain damage [62]. Another study showed that intravenous administration of aspartate aminotransferase [63], pyruvate and oxaloacetate [62,64] could significantly reduce glutamate levels in the blood in addition to accelerating the discharge of glutamate from the brain. These blood glutamate scavengers (BGS) work through decreasing intraparenchymal glutamate levels [59], significantly improving prognoses and outcomes [65], and extending the lifetimes of mice [38,66,67]. From this data, we conclude that the environments of the blood and brain have shared influences, and that blood glutamate is a significant factor in the brain.

## 7. Neurological Conditions Associated with BBB Permeability and Depression

Dysfunction of the glutamatergic system has been implicated in the pathophysiology of several neurological and neurogenerative disorders. There are several possible pathways through which BBB permeability affects depression. These include increases in CSF or S100B, a neurotrophic factor produced by astrocytes, which are both associated with depression, and increased permeability of the BBB [66,67,68]. In addition, studies have demonstrated that brain endothelial cells and tight junction proteins claudin-5 and occludin were disrupted after chronic stress, indicating weakened BBB integrity after stress. BBB permeability causes increased influx of peripheral material into the brain, leading to neuroinflammation and oxidative stress [69]. These outcomes all point to a correlation between BBB permeability and depression [70,71,72].

**Figure 2:**
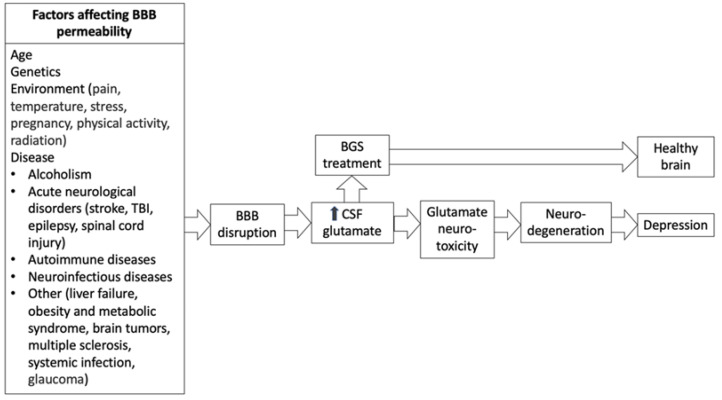
The relationship between conditions that disrupt the blood–brain-barrier, glutamate and depression.

Although the exact mechanisms of BBB permeability have not yet been clearly elucidated, BBB dysregulation has negative effects on a number of conditions, including stroke, TBI, Alzheimer’s disease, Parkinson’s disease, multiple sclerosis and schizophrenia [73,74], known to be a major contributor to the pathology of CNS disorders [75]. Specifically, glutamate excitotoxicity, in which cell death occurs as a result of excessive glutamate release from neurons and glial cells, contributes to the influx of glutamate in the BBB. Glutamate excitotoxicity is an acknowledged factor in neurological conditions [76]. From this evidence, it is likely that treatments that target BBB functionality may be a viable pathway to improving neurological outcomes [77] (Figure 2).

A full assessment of all neurodegenerative conditions associated with depression and BBB destruction is beyond the scope of our review. Here, we provide a preliminary examination of this relationship through examples of several conditions in which severe BBB destruction leading to an increase in brain extracellular glutamate levels in the short and long term has been well researched and documented.

### 7.1. Stroke

Stroke is the third most prevalent cause of mortality worldwide and can cause lifetime disability for survivors [78]. Ischemic stroke, the more common type of stroke, occurs after a thrombosis, embolism or systemic hypo-perfusion, which restrict blood flow to the brain. In an ischemic stroke, not enough oxygen and glucose are delivered to maintain cellular homeostasis. This results in cell death from a variety of causes, including from excitotoxicity. During stroke, the increase in BBB permeability is mainly due to changes in junctional protein expression and function [78,79,80]. This disruption begins immediately after vessel occlusion and continues for a significant amount of time after stroke.

The relationship between stroke and depression is also well documented. Post-stroke depression is an acknowledged symptom that affects recovery and mortality after the stroke incident, and early use of antidepressants has been shown to improve outcomes [79,81]. One preliminary study suggests that the use of the BGS pyruvate is a potential treatment for post stroke depression due to the interconnected relationship between glutamate, depression and the BBB [30].

### 7.2. TBI

The mechanisms of TBI, another major cause of disability and mortality, similarly involve glutamate dysregulation. Disruption of the BBB through inflammation causes cell death and brain edema [82], and TBI has a clear association with a breakdown in BBB permeability [80]. TBI, like stroke, has a known association with depression after its occurrence. Studies report that a quarter to a half of sufferers develop major depression within the first year following TBI [82,83]. A rodent model of TBI used pyruvate as an effective treatment toward mitigating post-TBI depressive-like symptoms, proposing its viability for future studies in the topic of treatments for post-TBI depression in humans [31].

### 7.3. Other

Other neurological conditions that are caused or exacerbated by BBB disruption and glutamate excitotoxicity and have an association with depression include Parkinson’s disease [84], Huntington’s disease [85], amyotrophic lateral sclerosis [86,87], multiple sclerosis [75,88], Alzheimer’s disease [89,90,91,92], epilepsy [93,94,95], aging [77] and schizophrenia [96,97]. There are likely interpolations to be made to similar conditions for which the association between BBB or depression lacks conclusive data [75], such as in hepatic encephalopathy [98] and brain tumors [99]. The idea of glutamatergic involvement in depression present during these conditions requires additional research.

## 8. Manipulation of Brain-Blood Glutamate Equilibrium

Excess brain glutamate can be mitigated by manipulating the brain-blood glutamate equilibrium and inducing excess glutamate from the brain’s interstitial fluid to flow into the body’s circulatory system [100]. A method proven in a rat model to reduce the early neuroanatomical and neurological signs caused by TBI includes treatment with BGS. This occurs by administering the enzymes glutamate-oxaloacetate transaminase 1 (GOT) or glutamate pyruvate transaminase (GPT) with their co-substrates oxaloacetic acid (OxAc) or pyruvate, respectively, which reduces excess brain glutamate by altering the balance between blood and brain glutamate [101,102,103]. This method does not directly stimulate or block synaptic NMDA receptors, avoiding some of the negative effects of direct interference. The ability of this method to mitigate emotional and cognitive deficits has begun to be investigated.

Studies indicate that low GOT levels are associated with poor neurological outcomes in humans after stroke, and high GOT levels are associated with a better neurological outcome [65,104]. GOT and GPT include glutamate as a substrate in the general formula: A + glutamate ← (enzyme) → C + D. In this formula, A represents the co-substrate; ← (enzyme) → symbolizes a reversible enzyme; and C and D are metabolites of the enzyme. These enzymes both use pyridoxal phosphate as a cofactor, and reversibly convert glutamate into 2-ketoglutarate, causing blood glutamate levels to reach levels below basal levels. Thus, in a state of impaired BBB permeability, and as a result of the equalization of glutamate levels between the extracellular cerebral fluid and plasma, much less glutamate is able to cross the BBB from the plasma into the extracellular cerebral fluid.

During glutamate transformation via an enzymatic reaction into 2-ketoglutarate, a buildup of 2-ketoglutarate occurs, leading to the possibility of the enzyme converting 2-ketoglutarate into glutamate. It is therefore beneficial to further break down 2-ketoglutarate to ensure the continual metabolism of glutamate. The enzyme 2-ketoglutarate dehydrogenase metabolizes 2-ketoglutarate through the general reaction: 2-ketoglutarate + lipoamide ← (2-ketoglutarate dehydrogenase) → S-succinyldihydrolipoamide + CO_2_ [105].

In addition to its success in limiting the excess glutamate and the neurological and immunological signs of traumatic injury to the brain and spinal cord, BGS effectively reduces the neurological and behavioral symptoms of subarachnoid hemorrhage [103] and middle cerebral artery occlusion [32]. BGS continues the physiological effects of glutamate in maintaining the metabolic and electrolyte balance and neuronal integrity and improving neuro-repair after brain injury. OxAC and GOT have been proposed as treatments for stroke [65,106]. The safety of OxAc has been demonstrated in elderly patients [107,108], and its potential to alleviate symptoms of anxiety, depression, suicidal ideation and aggression have been suggested in a clinical trial [109]. Thus, despite the destruction of the main mechanisms for maintaining glutamate homeostasis between the blood and the brain in diseases associated with impaired BBB, and a resultant increase in cerebral glutamate and the occurrence of mental disorders, the BGS method looks very promising for the treatment and prevention of depression, and in other conditions associated with high levels of brain glutamate.

Based on the mechanisms described above for the development of depression as a result of BBB disruption and glutamate neurotoxicity, there may be alternatives to BGS. Other therapeutic strategies are possible that would be aimed at restoring the BBB or facilitating the transport of glutamate through the BBB. The efficacy of BGS, however, has been proven in previous studies, which makes it a likely candidate for application in these circumstances as well.

## 9. Contrary Findings of Impaired BBB Permeability and Brain-Blood Glutamate Equilibrium

Despite the evidence for the involvement of glutamate in the pathophysiology of depression, a few contrary approaches appear to put this evidence in doubt. Studies using proton magnetic resonance spectroscopy (1H-MRS) have observed elevated regional levels of glutamatergic metabolites in patients with depression compared with controls [20]. However, other studies indicate inconsistent results, including increases [110], no differences [111,112] or decreases [113] in glutamatergic neurometabolite levels across different brain regions in patients with depression. This variability may be attributable to differences in the regions examined, MRS methodologies, stages or severities of illness or in medications such as antidepressant treatments [114].

This inconsistency can also be explained by the understanding that MRI usually cannot distinguish between a glutamate and glutamine peak and instead measures the combined glutamate-glutamine peak. Since glutamate breaks down into glutamine, this information does not reflect the state of increasing or decreasing levels of glutamate in the brain. In addition, most importantly, the results obtained using MRS are not very informative, since this technique cannot separate intracellular and extracellular brain glutamate. Therefore, the average result of total brain glutamate is given, including the blood found in that particular region.

This information does not reflect the level of extracellular glutamate that, according to the glutamate theory, is the cause of depression. Therefore, MRS studies of brain glutamate levels can be more confusing than illuminating, unlike glutamate concentration data obtained from CSF collection, microdialysis or brain slice staining for glutamate receptor counts.

Also, blood glutamate levels can be inconsistent, sometimes high and sometimes normal. Some data suggests that glutamate levels in plasma and whole blood are increased in patients with depression versus controls [17,115,116], while others have indicated no disparity between the two groups [16,117]. The reason for the observed elevation in blood glutamate levels have not yet been elucidated [34,38,118].

Blood cells also provide a source of blood glutamate. Cerebral infarction involves the release of platelets, which allows for the entrance of a large amount of glutamate into the blood [119]. Due to the secretion of glutamate in osteoclasts when stimulated with KCl or ATP, bone might be another source [120].

Additionally, blood and plasma glutamate levels can be recorded over a long timeframe and are highly dependent on gender [121,122], body temperature [123], diet [115], stress [116], meal time [124], age [118,119], background diseases [125] and even blood sampling sites [126]. Therefore, the level of blood/plasma glutamate probably plays an important role in the change in the level of brain glutamate, but the degree of destruction of the BBB plays a more important role. This ultimately determines the level of increase in brain glutamate and, as a consequence, the development of depression.

## 10. Conclusions

The development of antidepressant drugs has made much progress in the last several decades. However, standard treatment still relies exclusively on the monoaminergic system and therefore has many shortcomings. Existing monoaminergic antidepressants require long-term treatment of weeks or months to reach their full therapeutic efficacy and are effective in only a fraction of depressed patients. More nuanced analyses have revealed many factors and mechanisms that contribute to the development of MDD, including monoamine dysfunction, hormones, hypothalamic-pituitary-adrenal axis activation, genetics, neurogenesis, immune dysfunction and gender. In addition, the innovative theory about the involvement of the glutamate system aims to contribute more to our understanding of depression. In this review, we examined specific conditions in which depression is a co-morbidity that which are also associated with the glutamatergic system. In neurological and neurogenerative diseases with impaired BBB permeability and excitotoxicity as a contributing factor to the disease pathogenesis, we suggest that treatment for depression should be directed toward stabilizing the glutamate system through restoring homeostasis. In our opinion, drugs such as blood glutamate scavengers should be considered for the treatment of depression associated with these conditions. We anticipate more research on this topic will elucidate this and other treatment options.

## Data Availability

Not applicable.
